# Three-dimensional black-blood multi-contrast carotid imaging using compressed sensing: a repeatability study

**DOI:** 10.1007/s10334-017-0640-1

**Published:** 2017-06-26

**Authors:** Jianmin Yuan, Ammara Usman, Scott A. Reid, Kevin F. King, Andrew J. Patterson, Jonathan H. Gillard, Martin J. Graves

**Affiliations:** 1 Department of Radiology, School of Clinical Medicine, University of Cambridge, Level 5, Box 218, Addenbrooke’s Hospital, Hills Rd, Cambridge, CB2 0QQ UK; 2 GE Healthcare, Amersham, UK; 3 GE Healthcare, Waukesha, WI USA; 4 Department of Radiology, Cambridge University Hospitals NHS Foundation Trust, Cambridge, UK

**Keywords:** Multi-contrast, Carotid MRI, Compressed sensing

## Abstract

**Objective:**

The purpose of this work is to evaluate the repeatability of a compressed sensing (CS) accelerated multi-contrast carotid protocol at 3 T.

**Materials and methods:**

Twelve volunteers and eight patients with carotid disease were scanned on a 3 T MRI scanner using a CS accelerated 3-D black-blood multi-contrast protocol which comprises *T*_1_w, *T*_2_w and PDw without CS, and with a CS factor of 1.5 and 2.0. The volunteers were scanned twice, the lumen/wall area and wall thickness were measured for each scan. Eight patients were scanned once, the inter/intra-observer reproducibility of the measurements was calculated.

**Results:**

In the repeated volunteer scans, the interclass correlation coefficient (ICC) for the wall area measurement using a CS factor of 1.5 in PDw, *T*_1_w and *T*_2_w were 0.95, 0.81, and 0.97, respectively. The ICC for lumen area measurement using a CS factor of 1.5 in PDw, *T*_1_w and *T*_2_w were 0.96, 0.92, and 0.96, respectively. In patients, the ICC for inter/intra-observer measurements of lumen/wall area, and wall thickness were all above 0.81 in all sequences.

**Conclusion:**

The results show a CS accelerated 3-D black-blood multi-contrast protocol is a robust and reproducible method for carotid imaging. Future protocol design could use CS to reduce the scanning time.

## Introduction

High-resolution magnetic resonance imaging (MRI) is a useful clinic method to assess carotid plaque vulnerability, due to its excellent soft tissue contrast [1, 2]. A multi-contrast MR protocol comprising PDw, *T*_1_w and *T*_2_w sequences can identify high-risk intraplaque components with high sensitivity and specificity [2]. Though still not used in clinical routine, the blood suppressed multi-contrast carotid MRI protocol has been used in previous research studies for plaque classification [2], component segmentation [3, 4] and also to determine the age of intraplaque hemorrhage [5].

Black-blood techniques have been used to suppress the blood signal in the lumen to improve vessel wall visualisation. This is usually achieved in 2-D imaging with the use of magnetisation preparation schemes such as double [6, 7], or quadruple inversion-recovery [8]. For 3-D imaging, non-selective preparation schemes such as motion-sensitized driven-equilibrium (MSDE) [9, 10] or delay alternating with nutation for tailored excitation (DANTE) [11, 12] have been used. Volumetric (3-D) readout based on either fast-spin-echo [3, 13] or gradient-echo [12] allows the acquisition of near isotropic voxels, which could potentially improve the plaque component quantifications. However, one of the disadvantages of 3-D protocols in previous carotid studies is the long acquisition time (approximately 20–45 min), which could lead to poor patient compliance.

Several techniques have been introduced to reduce the 3-D acquisition time. Parallel imaging (PI) methods have been introduced, with the use of multi-channel coils [6, 14]. One of the studies has shown its ability to reduce the acquisition time by a factor of two whilst maintaining a high reproducibility for plaque quantitative measurement [6]. Dedicated pulse sequences have also been developed to acquire multiple contrast weightings in a single acquisition, such as the multi-contrast atherosclerosis characterization (MATCH) sequence, which can acquire hyper *T*_1_w, gray blood, and *T*_2_w images in a single 5-min sequence [15]. More recently, the method of compressed sensing (CS) has been introduced into MRI as an alternative method to accelerate image acquisition [16], and has been applied to carotid imaging [17–19]. These studies showed that the CS accelerated MERGE (motion-sensitizing driven equilibrium prepared 3-D rapid gradient echo) sequence could be used for rapid 3-D carotid wall imaging. Whilst the first two techniques (PI and MATCH) have been used for multi-contrast purposes, the use of CS has not previously been validated in a multi-contrast protocol. The purpose of this work, therefore, is to evaluate the use of CS within a current standard carotid multi-contrast protocol. Volunteer scans were carried out to evaluate the interscan reproducibility and patients with carotid atherosclerotic diseases were recruited to validate inter/intra-observer reproducibility of the morphology measurements based on the CS accelerated sequences.

## Materials and methods

### Study subjects

This study had ethical approval and informed consent was obtained from each volunteer and patient. Twelve volunteers (eight men, mean age 34, range 24–55 years) and eight patients (four men, mean age 75, range 72–87 years) with a carotid artery stenosis greater than 50% on duplex ultrasound were scanned on a 3 T MRI system (MR 750, GE Healthcare, Waukesha, WI), using a four-channel phased-array neck coil (PACC, MachNet, Roden, The Netherlands). To evaluate the interscan reproducibility of the sequences, the 12 volunteers were scanned for a second time using the same protocol. The time interval between the two scans was 14 days (range 7–28 days).

### Compressed sensing

The compressed sensing was achieved by using a Gaussian pseudo-random distribution undersampling pattern in *k*-space. The 32 × 32 area in the *k*-space center remained fully sampled to achieve high image quality. During the image reconstruction, the following objective function was applied:

1 $$\hat{m}\; = \;\left\| {\varPsi m} \right\| _{1} {\text{such that}} \left\| {F\hat{m} - y} \right\|_{2}^{2} \le \varepsilon ,$$

where $\psi$ is the sparsifying transform, which uses the gradient transform implemented as a nearest neighbor finite difference of the complex image *m*, $F$ is the fourier transform operator and $y$ is the acquired *k*-space data [16]. Fifteen iteration loops were used to minimize the penalty (L1-norm). In each of the iterations, the acquired *k*-space was subtracted back into the estimated *k*-space to maintain the data consistency. The CS sampling and reconstruction algorithm also enabled the combined use of parallel imaging, with the auto-calibrated reconstruction of the Cartesian data (ARC) [20] method, in order to reduce the overall examination time for both volunteers and patients. The investigation for the effects of varying CS acceleration factors alone, i.e., without the use of ARC, was evaluated using the *T*_1_w acquisition. For the PDw and *T*_2_w acquisitions, the CS was combined with an ARC acceleration of two in the phase encoding direction. The ARC and CS were combined sequentially with the CS algorithm applied as the first step and ARC as the second step. Lastly, a sum-of-squares coil combination was performed to get the final image. Further details of the image acquisition and reconstruction can be found in the references [21–23].

### Imaging protocol

All the subjects were imaged using a multi-contrast protocol listed in Table 1. Except for the patient scan, only a CS factor of 1.5 in PDw and *T*_2_w was used due to limited scanning time. Coronal imaging slabs of the 3-D sequences were centered at the carotid bifurcation.

**Table 1:** Scanning parameters for the multi-contrast protocol

Contrast	Time-of-flight	*T*_1_w	PDw	*T*_2_w
Sequence	3-D SPGR	3-D FSE	3-D FSE	3-D FSE
Acquisition direction	Axial	Coronal	Coronal	Coronal
Blood suppression	–	DANTE	iMSDE	iMSDE
Echo time (ms)	2.2	16.9	21.6	51.6
Repetition time (ms)	5.9	540	2000	2000
Flip angle (°)	20	Variable flip angle	Variable flip angle	Variable flip angle
FOV (mm^3^)	140 × 140 × 64	140 × 140 × 67	140 × 140 × 56	140 × 140 × 56
Acquisition matrix	256 × 256 × 32	224 × 224 × 48	224 × 224 × 40	224 × 224 × 40
ARC Parallel Imaging (phase × slice)	–	–	2 × 1	2 × 1
CS acceleration	Non-CS	Non-CS/1.5/2.0	Non-CS/1.5/2.0	Non-CS/1.5/2.0
Acquisition time	1:35	3:16/2:13/1:41	3:36/2:36/2:07	3:36/2:36/2:07

*T*_1_w images were acquired by a DANTE [11] prepared 3-D FSE sequence. Images were acquired without CS acceleration, and with CS acceleration factor of 1.5 and 2.0. The scanning times were 3 min 16 s, 2 min 13 s and 1 min 41 s, respectively. The parameters for DANTE preparation were: the number of pulses: 150; 3 G/cm; gradient axes: *X*, *Y* and *Z*; flip angle: 13°; DANTE pulse repetition time: 1 ms. No parallel imaging was used for the *T*_1_w images. *T*_2_w and PDw images were acquired using an iMSDE [9] prepared 3-D FSE sequence. The first-order moment (*m*_1_) was empirically set to 412 mTms^2^/m. The scanning time was 3 min 36 s, 2 min 36 s and 2 min 7 s for both of the sequences without CS, and with CS factors of 1.5 and 2.0. The CS and ARC were combined in a sequential way [23]. Fat suppression was performed using an Adiabatic SPectral Inversion Recovery (ASPIR) pulse. The acquired resolution for *T*_1_w, *T*_2_w and PDw was 0.6 × 0.6 × 1.4 mm^3^. Electrocardiography (ECG) gating was not used in the protocol.

### Image analysis

The acquired multi-contrast images were first reformatted into the axial plane, and then interpolated into a voxel size of 0.2 × 0.2 × 0.3 mm^3^, using MATLAB (The MathWorks, Inc., Natick, MA). Carotid artery lumen and outer wall boundaries were manually drawn by an experienced observer who has more than 2 years’ experience in carotid imaging, using a Food and Drug Administration (FDA) proved DICOM viewer (OsiriX 5.5.2, Pixmeo, Geneva, Switzerland).

For the volunteer images, five contiguous slices in the common, internal and external carotid artery (CCA, ICA and ECA), 5 mm below and 5 mm above the bifurcation were used for morphological measurements. The lumen area was defined as the area inside the lumen contour, and the wall area was defined as the area between the outer wall boundary and inner lumen contour. The mean radius of the lumen and outer wall was calculated by simplifying the geometry as a circle with the same area. The mean wall thickness was determined as the difference between lumen radius and outer wall radius. The wall-lumen sharpness was also calculated for each CS factor in the *T*_1_w sequences [24]. To calculate the wall-lumen sharpness, a line profile perpendicular from the vessel wall to the lumen was analysed. Due to the blood suppression, the lowest signal pixel (in the blood) in the line profile is set to zero and the highest intensity pixel (in the wall) is set to be 1. The distance between the pixel intensity at 0.2 and 0.8 was calculated. The image sharpness was defined as one over the distance. For the patient images, the vessel wall and lumen boundaries were manually drawn on each of the slices containing atherosclerotic plaque. The plaque was defined as a focal wall thickness ≥1.5 mm [25]. To test the intra-observer reproducibility, the *T*_1_w, *T*_2_w and PDw images with a CS factor of 1.5 were analysed twice by the same observer. To test the inter-observer reproducibility, the *T*_1_w, *T*_2_w and PDw images were analysed by a second observer who also has more than 2 years of carotid imaging, and the results were compared with the first observer. Both of the observers made their measurements independently for each time, and were blinded to the subjects’ clinical information.

### Statistical analysis

Interclass correlations (ICCs) and coefficient of variation (CoV) were calculated to evaluate the agreement of wall/lumen area and wall thickness measurements between two volunteer scans. The CoV is defined as the ratio of the standard deviation of the difference between the two measurements to the mean value

2 $${\text{CoV}}\; = \;\frac{{{\text{sd}}\left( {x_{1i} - x_{2i} } \right)}}{{\left( {\mathop \sum \nolimits_{i = 1}^{n} \frac{{x_{1i} + x_{2i} }}{2}} \right)/n}},$$

where *x*_1_ and *x*_2_ are the first and second measurement, *n* is the number of subjects, *i* = 1, 2,… *n* and sd is the standard deviation.

The measurement differences from *T*_1_w images between two scans were analysed using the Bland–Altman method. The inter/intra-observer variabilities from patients’ scans were also evaluated using the ICC and CoV. An ICC value above 0.75 was considered as excellent agreement. The 0.40–075 was good agreement and below 0.40 was poor agreement. A two-tailed paired student’s *t* test was used to compare the wall thickness measurements between non-CS and CS accelerated *T*_1_w sequences in patients. Statistical significance was defined if *p* < 0.05. Continuous data were presented as mean ± sd. The statistical analysis was performed using R (version 3.2.2).

## Results

All of the twelve volunteers completed the scans. Seven out of eight patients completed the scan. One patient did not finish the scan due to discomfort.

**Fig. 1:**
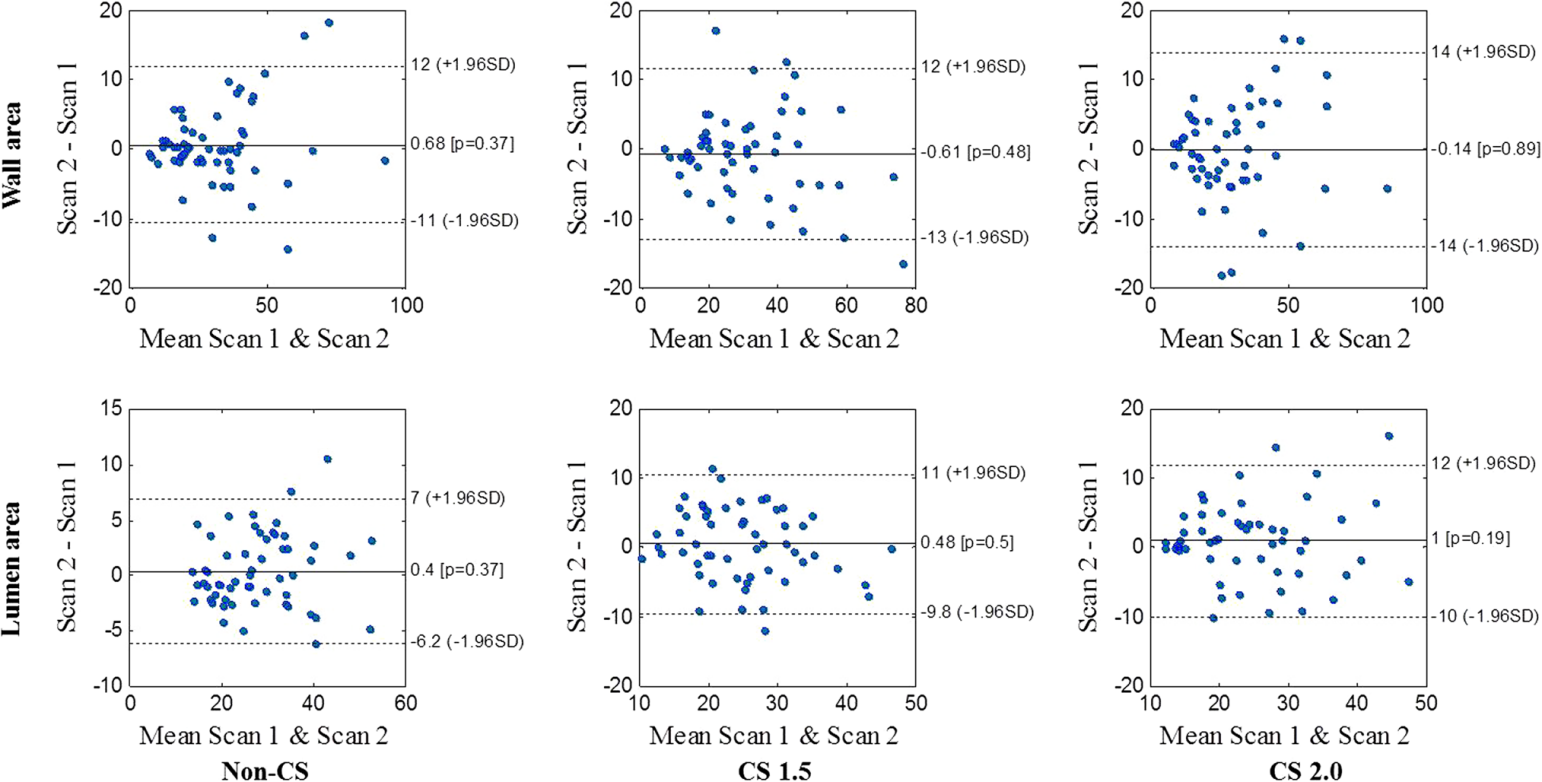
Bland-Altman plots of volunteer lumen and wall area measurements from *T*_1_w sequences between two repeated scans (all units mm^2^)

**Fig. 2:**
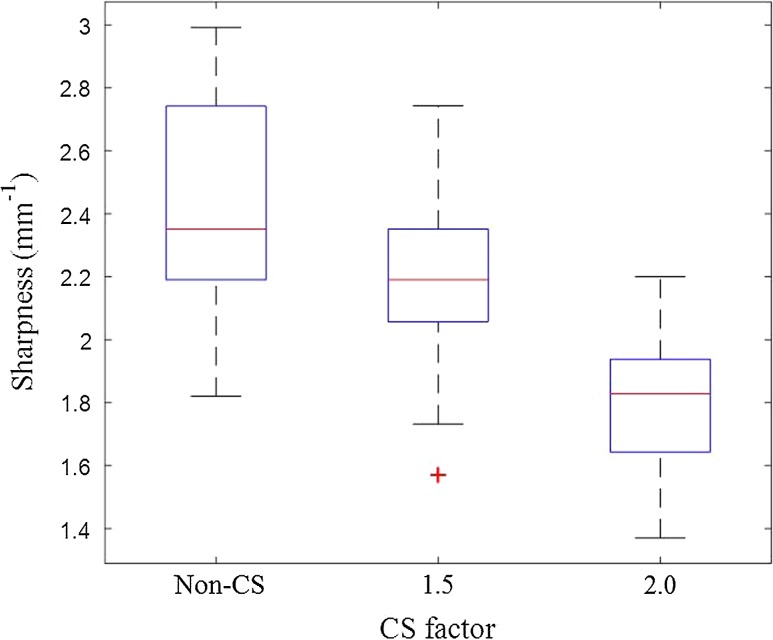
The wall-lumen sharpness measured from volunteer *T*_1_w images with different CS factors

Figure 1 shows the Bland–Altman plots of lumen and wall area measurement of volunteer *T*_1_w images between two scans with non-CS, CS factor of 1.5 and 2.0. The ICC (95% CI) and CoV for lumen and wall area measured from the repeated volunteer scans using the three multi-contrast sequences with different CS factors are shown in Table 2. Excellent correlation of wall/lumen area measurement were found between two scans (all ICCs >0.80). Figure 2 shows the wall-lumen sharpness calculated from the *T*_1_w volunteer images. The sharpness decreases with increasing CS factor.

**Table 2:** ICC (95% CI) and CoV of the scan-rescan measurements of the multi-contrast sequences using no CS, CS factor of 1.5 and 2.0 from volunteers

	No CS	CS 1.5	CS 2.0
ICC			
*T*_1_w			
Wall area	0.94 (0.90–0.97)	0.81 (0.69–0.88)	0.81 (0.70–0.89)
Lumen area	0.95 (0.91–0.97)	0.92 (0.87–0.95)	0.91 (0.85–0.95)
* T*_2_w			
Wall area	0.96 (0.94–0.97)	0.97 (0.94–0.98)	0.81 (0.74–0.86)
Lumen area	0.97 (0.93–0.98)	0.96 (0.94–0.98)	0.91 (0.81–0.96)
PDw			
Wall area	0.91 (0.79–0.97)	0.95 (0.92–0.97)	0.82 (0.80–0.92)
Lumen area	0.95 (0.90–0.96)	0.96 (0.92–0.97)	0.91 (0.81–0.95)
CoV			
* T*_1_w			
Wall area	12%	26%	24%
Lumen area	11%	16%	17%
* T*_2_w			
Wall area	13%	14%	20%
Lumen area	14%	15%	17%
PDw			
Wall area	19%	14%	22%
Lumen area	12%	14%	18%

**Fig. 3:**
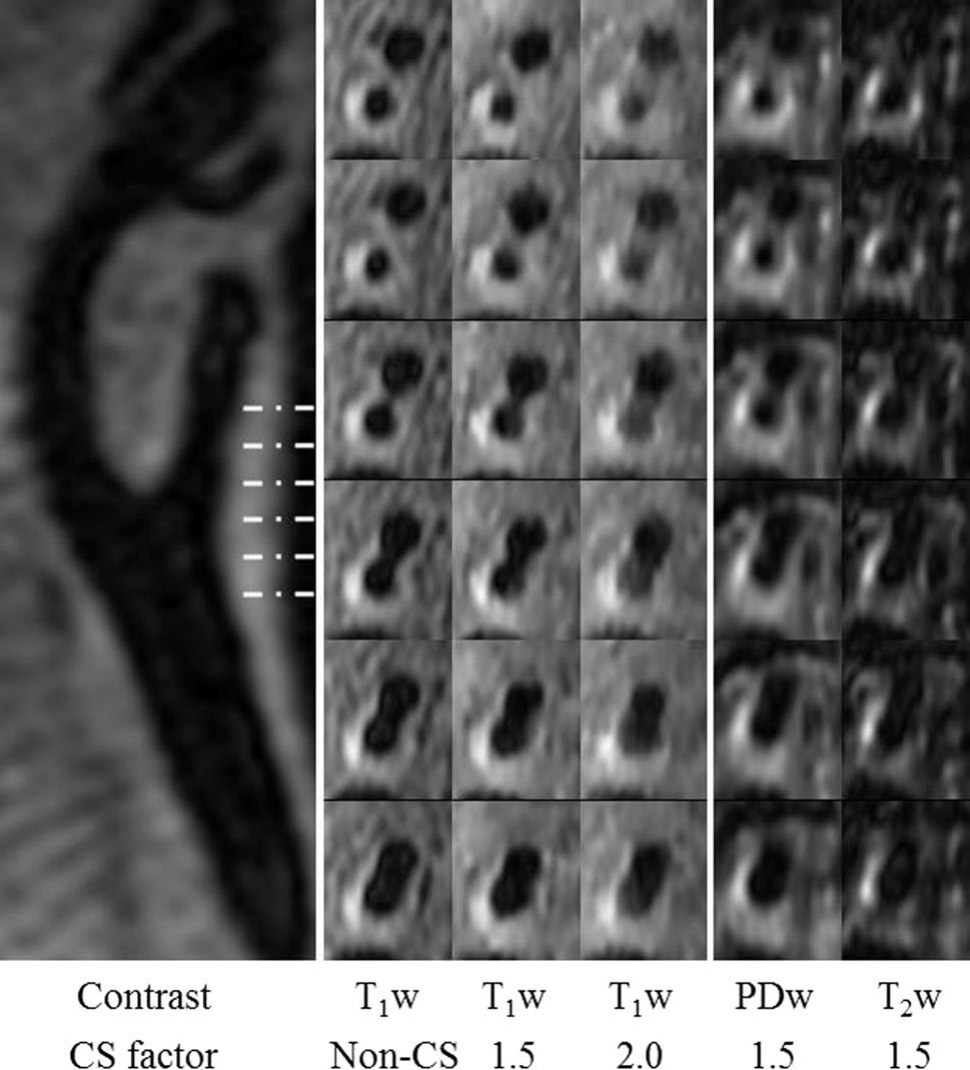
Multi-contrast carotid images of a 80-year-old male. The plaque contains an intraplaque haemorrhage

**Fig. 4:**
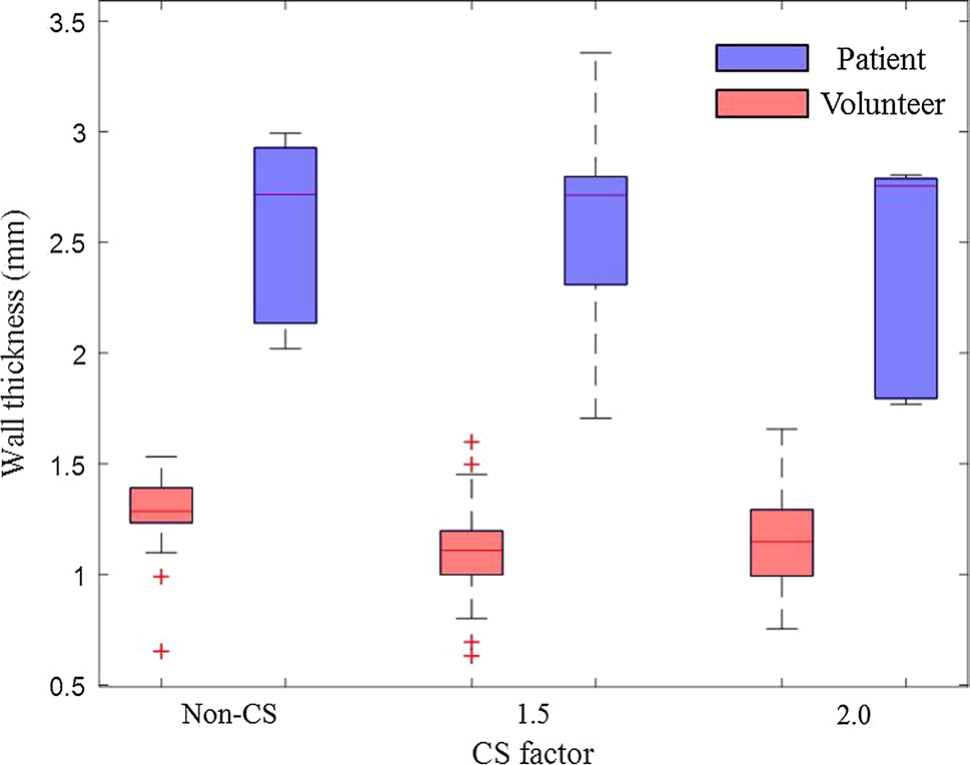
Volunteer and patient’s wall thickness at different CS factors of *T*_1_w images

Figure 3 shows an example of a patient image with intraplaque hemorrhage. From the *T*_1_w images, the wall-lumen and outer wall boundaries become blurred with increasing CS factor. However, for the wall thickness measurement, there are no significant differences comparing the CS accelerated sequence with the non-CS sequence (Non-CS vs. CS1.5: 2.43 ± 0.57 mm vs. 2.58 ± 0.87 mm, *p* = 0.58; Non-CS vs. CS2.0: 2.43 ± 0.57 mm vs. 2.39 ± 0.85 mm, *p* = 0.59). When compared to the volunteers, the patients have a thicker wall thickness (*p* < 0.05), as shown in Fig. 4.

Table 3 shows the ICC with 95% CI and CoV of the intra/inter-observer reproducibility in measuring the lumen/wall area and wall thickness in multi-contrast sequences with a CS factor of 1.5 from the patients. All the ICCs were above 0.81. Table 4 shows the intra-observer reproducibility of three different CS factors using *T*_1_w sequence in patient scans. The ICCs were above 0.83.

**Table 3:** ICC (95% CI) and CoV for the intra/inter-observer measurements of the multi-contrast protocol using CS factor of 1.5 from patients’ scans

	PDw	*T*_1_w	*T*_2_w
ICC			
Intra-observer			
Wall area	0.86 (0.51–0.98)	0.84 (0.50–0.96)	0.82 (0.43–0.97)
Lumen area	0.98 (0.95–0.99)	0.98 (0.92–1.00)	0.98 (0.96–0.99)
Wall thickness	0.98 (0.96–0.99)	0.86 (0.57–0.96)	0.97 (0.95–0.99)
Inter-observer			
Wall area	0.84 (0.50–0.96)	0.87 (0.40–0.98)	0.86 (0.51–0.97)
Lumen area	0.98 (0.95–0.99)	0.95 (0.77–0.99)	0.98 (0.93–0.99)
Wall thickness	0.98 (0.94–0.99)	0.92 (0.63–0.97)	0.95 (0.89–0.99)
CoV			
Intra-observer			
Wall area	22%	25%	27%
Lumen area	13%	14%	12%
Wall thickness	12%	19%	14%
Inter-observer			
Wall area	25%	17%	20%
Lumen area	13%	15%	14%
Wall thickness	14%	16%	16%

**Table 4:** ICC (95% CI) and CoV for the intra-observer measurements of the *T*_1_w sequences using no CS, CS factor of 1.5 and 2.0 from patients’ scans

	No CS	CS 1.5	CS 2.0
ICC			
Wall area	0.95 (0.79–0.99)	0.84 (0.50–0.96)	0.89 (0.54–0.98)
Lumen area	0.99 (0.95–1.00)	0.98 (0.92–1.00)	0.93 (0.66–0.99)
Wall thickness	0.90 (0.60–0.98)	0.86 (0.57–0.96)	0.93 (0.67–0.99)
CoV			
Wall area	15%	25%	19%
Lumen area	11%	14%	18%
Wall thickness	14%	19%	13%

## Discussion

This study demonstrates, for the first time, the usefulness of CS acceleration in a multi-contrast black-blood carotid protocol to reduce the overall acquisition time at 3 T. The results show that CS accelerated sequences have a good scan-rescan reproducibility in carotid morphological measurement in volunteers, and good inter/intra-observer reproducibility of morphological measurement in patients.

Multi-contrast MRI has been used widely in assessing plaque components and vulnerability [2, 3, 12, 26–34]. Originally this was performed at 1.5 T [2, 26–30], however the studies are now more commonly performed at 3 T due to the superior signal-to-noise ratio (SNR) and contrast-to-noise ratio (CNR) [31–33, 35–37]. In addition, the development of 3-D sequences allows for larger coverage, better through-plane resolution, higher scanning efficiency, less motion artefact and more precise plaque segmentation [3, 12, 15]. Whilst acceleration techniques such as PI have been introduced and validated [6], the use of CS has not previously been validated for the multi-contrast protocol. The CS technique has previously been used in carotid studies to reduce the acquisition time in sequences such as MERGE [17–19, 38] and black-blood dynamic contrast enhanced MRI [39]. The studies showed that CS can be used to (1) reduce the acquisition time without significantly impacting on the diagnostic quality, (2) reduce motion artefacts and (3) achieve higher temporal resolution in dynamic imaging. Previous studies have also demonstrated that CS produces better image quality than PI [40], and that the combination of CS and PI can achieve even better image quality than either of the techniques used alone [41]. This study has, for the first time, reported the scan-rescan reproducibility and intra-inter observer repeatability of carotid morphological measurement in a multi-contrast protocol. The results showed that wall area, lumen area and wall thickness is reproducible in a CS accelerated multi-contrast protocol, using a productised reconstruction with accepted reconstruction time.

The results from this study show that the wall-lumen sharpness decreases (Fig. 2), and the CoV of volunteer scan-rescan measurements increases with increasing CS factor (Table 2). This indicates that the use of CS brings additional uncertainty in the morphological measurement. Table 2 indicates that with either no or small CS factors (CS 1.5), the CoV of morphological measurements in repeatability scans is acceptably low.

There are several limitations in this study. Firstly, the number of patients is quite small. Only eight patients were scanned using the current multi-contrast protocol. Therefore, only limited plaque components and examples were investigated. However, good reproducibility of the quantitative analysis from the volunteer scan demonstrates the feasibility of applying the CS-accelerated protocol for a future large-scale patient study. The second limitation is that a coronal acquisition with anisotropic resolution was used in this study as a trade-off between blood suppression, image SNR, scanning time and coverage. This may lead to limitations in the wall thickness measurements and potentially affect the plaque characterization in the reconstructed axial image. Nevertheless, the result from the current scanning settings showed good repeatability of the wall thickness measurements. Future optimisation should consider the use of isotropic resolution acquisitions for better plaque characterization. The third limitation is that the CS reconstruction method used in this study was a vendor provided on-line algorithm, which was optimised for clinically acceptable reconstruction times. Further optimisation of the acquisition and reconstruction parameters, or to even use the multi-contrast sequences for joint reconstruction [42], could potentially improve the image quality, albeit probably at the expense of increased reconstruction time. Fourthly, considering the limited scanning time in practice, ARC acceleration of 2 × 1 and only a single CS factor of 1.5 was used for PDw and *T*_2_w sequences. The results from the *T*_1_w may not necessarily be extrapolated to these two contrast weighted images. The last limitation is that a fixed echo train length was used in this study, as we primarily considered the CS as an additional feature to the existing optimised protocol setup. Longer echo train length with optimised flip angle schemes could further reduce the acquisition time, but this is outside the scope of this study.

## Conclusion

In conclusion, this study shows that the current 3-D carotid black-blood multi-contrast protocol could be accelerated by CS, in addition to PI, with robust and reproducible morphology measurements. With the current protocol setup, a CS factor of 1.5 in combination with a parallel imaging acceleration factor of two can be used for a multi-contrast protocol.
